# Pathological Internet Use and Risk-Behaviors among European Adolescents

**DOI:** 10.3390/ijerph13030294

**Published:** 2016-03-08

**Authors:** Tony Durkee, Vladimir Carli, Birgitta Floderus, Camilla Wasserman, Marco Sarchiapone, Alan Apter, Judit A. Balazs, Julio Bobes, Romuald Brunner, Paul Corcoran, Doina Cosman, Christian Haring, Christina W. Hoven, Michael Kaess, Jean-Pierre Kahn, Bogdan Nemes, Vita Postuvan, Pilar A. Saiz, Peeter Värnik, Danuta Wasserman

**Affiliations:** 1 National Centre for Suicide Research and Prevention of Mental Ill-Health (NASP), Karolinska Institutet, Stockholm SE-17177, Sweden; vladimir.carli@ki.se (V.C.); danuta.wasserman@ki.se (D.W.); 2 Department of Clinical Neuroscience, Karolinska Institutet, Stockholm SE-17177, Sweden; birgitta.floderus@ki.se; 3 Department of Medicine and Health Science, University of Molise, Campobasso 86100, Italy; camillaw@gmail.com (C.W.); marco.sarchiapone@gmail.com (M.S.); 4 Department of Child and Adolescent Psychiatry, New York State Psychiatric Institute, Columbia University, New York, NY 10032, USA; ch42@cumc.columbia.edu; 5 National Institute for Migration and Poverty, Via San Gallicano, Roma 25/A, Italy; 6 Feinberg Child Study Centre, Schneider Children’s Medical Centre, Tel Aviv University, Tel Aviv 49202, Israel; asapter@gmail.com; 7 Vadaskert Child and Adolescent Psychiatric Hospital, Budapest 1021, Hungary; judit.agnes.balazs@gmail.com; 8 Institute of Psychology, Eötvös Loránd University, Budapest 1064, Hungary; 9 Department of Psychiatry, Center for Biomedical Research in the Mental Health Network (CIBERSAM), University of Oviedo, Oviedo 33006, Spain; bobes@uniovi.es (J.B.); pilaralejandra.saiz@gmail.com (P.A.S.); 10 Section for Disorders of Personality Development, Clinic of Child and Adolescent Psychiatry, Centre of Psychosocial Medicine, University of Heidelberg, Heidelberg 69115, Germany; romuald.brunner@uni-heidelberg.de (R.B.); Michael.kaess@uni-heidelberg.de (M.K.); 11 National Suicide Research Foundation, Western Rd., Cork, Ireland; pcorcoran@ucc.ie; 12 Department of Clinical Psychology, Iuliu Hatieganu University of Medicine and Pharmacy, Str. Victor Babes Nr. 8, Cluj-Napoca 400000, Romania; doina_octaviancosman@yahoo.com (D.C.); nemes_bogdan@yahoo.com (B.N.); 13 Research Division for Mental Health, University for Medical Information Technology (UMIT), Klagenfurt, Innsbruck 6060, Austria; Christian.haring@tilak.at; 14 Department of Epidemiology, Mailman School of Public Health, Columbia University, New York, NY 10032, USA; 15 Department of Psychiatry, Centre Hospitalo-Universitaire de Nancy, Université de Lorraine, Nancy, Vandoeuvre-lès-Nancy 54500, France; jp.kahn@chu-nancy.fr; 16 Slovene Center for Suicide Research, Andrej Marušič Institute, University of Primorska, Koper 6000, Slovenia; vita.postuvan@upr.si; 17 Centre of Behavioral and Health Sciences, Estonian-Swedish Mental Health & Suicidology Institute, Tallinn University, Tallinn 10120, Estonia; peeterv@suicidology.ee

**Keywords:** pathological Internet use, Internet addiction, risk-behavior, multiple risk-behaviors, unhealthy lifestyles, adolescents, SEYLE

## Abstract

Risk-behaviors are a major contributor to the leading causes of morbidity among adolescents and young people; however, their association with pathological Internet use (PIU) is relatively unexplored, particularly within the European context. The main objective of this study is to investigate the association between risk-behaviors and PIU in European adolescents. This cross-sectional study was conducted within the framework of the FP7 European Union project: Saving and Empowering Young Lives in Europe (SEYLE). Data on adolescents were collected from randomized schools within study sites across eleven European countries. PIU was measured using Young’s Diagnostic Questionnaire (YDQ). Risk-behaviors were assessed using questions procured from the Global School-Based Student Health Survey (GSHS). A total of 11,931 adolescents were included in the analyses: 43.4% male and 56.6% female (M/F: 5179/6752), with a mean age of 14.89 ± 0.87 years. Adolescents reporting poor sleeping habits and risk-taking actions showed the strongest associations with PIU, followed by tobacco use, poor nutrition and physical inactivity. Among adolescents in the PIU group, 89.9% were characterized as having multiple risk-behaviors. The significant association observed between PIU and risk-behaviors, combined with a high rate of co-occurrence, underlines the importance of considering PIU when screening, treating or preventing high-risk behaviors among adolescents.

## 1. Introduction

Adolescence is a transitional period characterized by considerable changes in physical, social and psychological attributes [1]. Moreover, relationships with peers, family and society undergo distinct changes during this transient period, as adolescents begin to assert autonomy over their decisions, emotions and behaviors [2]. Social aptitudes in adolescents often develop in the course of psychosocial interactions within different learning contexts [3]. Given the extensive platform for fostering social cognition and interpersonal skills [4,5], the Internet has proven to be a new and unique channel for psychosocial development among adolescents [6,7]. 

Despite these inherent advantages, studies have shown that frequent and prolonged use of online applications has the propensity to displace conventional social interactions and relationships [8,9]. There is evidence demonstrating that accumulative time online displaces time on face-to-face interaction with family and friends [10], participating in extra-curricular activities [11], completing academic tasks [12], proper eating habits [13], physical activity [14] and sleeping [15]. As adolescents are spending more time online, there is a risk that their Internet use can become excessive or even pathological [16]. 

Pathological Internet use (PIU) is characterized by excessive or poorly-controlled preoccupations, urges or behaviors regarding Internet use that lead to impairment or distress [17]. PIU has conceptually been modelled as an impulse-control disorder and classified as a taxonomy of behavioral addiction akin to the nature of pathological gambling [18]. Despite recent advancements in PIU research, efforts to understand this phenomenon are impeded by the lack of international consensus on the diagnostic criteria of the condition. It is neither listed in the Diagnostic and Statistical Manual of Mental Disorders (DSM) nor the International Classification of Diseases (ICD) nosological systems. The major challenge facing PIU research is its conception as an addictive disorder.

In light of these contentions, the recently-published DSM-5 [19] has included behavioral addiction (non-substance-related addictive disorders) as an official diagnostic category, with gambling disorder (GD) being the only condition listed in this new classification. Internet gaming disorder (IGD) is also a potential subtype of behavioral addiction that was considered for inclusion in the DSM nosological system; however, evidence supporting IGD as a diagnostic disorder was still lacking. IGD was subsequently included into Section III of the DSM-5, as a condition that required further study [20], in order to determine its eventual suitability as a diagnostic disorder. In spite of the current nosological ambiguity of PIU, there continues to be surmounting evidence showing a strong link between PIU and other forms of addiction [21,22,23,24]. 

Research shows that individuals with PIU share neurological, biological and psychosocial attributes with both behavioral and substance-related addictions [25,26,27,28,29]. Based on a theoretical model denoted by Griffiths [30], there are six core symptoms exhibited in addictive disorders that are applicable to PIU. These include: salience (preoccupation with online activities), mood modification (using the Internet to escape or alleviate stress), tolerance (necessity to stay online longer), withdrawal (depression and irritability when offline), conflicts (interpersonal and intrapsychic) and relapse (failed attempts to discontinue Internet use). These core components provide a theoretical framework for estimating the magnitude of PIU. 

Prevalence rates for PIU vary considerably across countries, partly due to the heterogeneity of its definition, nomenclature and diagnostic assessment. In an effort to estimate a global prevalence, Cheng and Li [31] addressed these discrepancies by applying a random effects meta-analysis using studies with comparable psychometric instruments and criteria. This approach yielded a total of 89,281 participants from 31 countries spanning across several world regions. Results showed that the global prevalence of PIU was 6.0% (95% CI: 5.1–6.9) with only moderate heterogeneity. 

Prevalence studies assessing PIU at the European level using representative samples are limited. Despite this paucity, there is emerging epidemiological evidence indicating stable trends in prevalence rates among this target group. In a representative sample of European adolescents (n = 18,709) aged 11–16 years, Blinka *et al*. [32] showed that the prevalence of PIU was 1.4%. This coincides with rates reported by Tsitsika *et al*. [33], who estimated a PIU prevalence of 1.2% in a representative sample of European youth (n = 13,284) aged 14–17 years. Durkee and colleagues [34], however, observed a slightly higher PIU prevalence of 4.4% in a representative sample of European adolescents (n = 11,956) aged 14–16 years. Prevalence rates for PIU in Europe were shown to be significantly higher in males than females, increase with age, differ by country and linked with an array of mental and behavioral disorders [35,36,37,38,39].

The onset of risk-behaviors frequently occurs during adolescence with a high likelihood of continuity into adulthood. Males tend to have a higher prevalence than females, and the frequency of risk-behaviors tends to increase with age [40]. There are distinct levels of severity ranging from low-risk (poor sleeping habits, poor nutrition and physical inactivity) to high-risk (excessive alcohol use, illicit drug use and tobacco use) behaviors. Research has typically assessed risk-behaviors as independent entities, albeit that clear evidence shows their co-occurrence, even at an early age [41,42]. Populations with multiple risk-behaviors have the greatest risk for chronic diseases, psychiatric disorders, suicidal behaviors and premature death compared to individuals with single or no risk-behaviors [43,44]. Given the concurrent nature of risk-behaviors, it is imperative to understand their implication on adolescents’ risk of PIU.

The Youth Risk Behavior Surveillance System (YRBSS) in the U.S. ascertains that risk-behaviors are a major contributor to the leading causes of morbidity among adolescents and young people [45]. Apart from this implicit supposition, there is relatively little research that systematically scrutinizes the extent to which these forms of behavior relate to adolescent PIU, particularly within the European context. Epidemiological investigations are necessary in order to acquire a better understanding of this phenomenon.

Based on a large, representative sample of school-based adolescents in Europe, the primary objective of this study is to investigate the association between risk-behaviors (*i.e.*, alcohol use, illicit drug use, tobacco use, risk-taking actions, truancy, poor sleeping habits, poor nutrition and physical inactivity) and distinct forms of Internet use. 

## 2. Materials and Methods

### 2.1. Study Design and Population

The present cross-sectional study was performed within the framework of the European Union project: Saving and Empowering Young Lives in Europe (SEYLE) [46]. Adolescents were recruited from randomly-selected schools across study sites in Austria, Estonia, France, Germany, Hungary, Ireland, Israel, Italy, Romania, Slovenia and Spain, with Sweden serving as the coordinating center. 

The inclusion criteria for selecting eligible schools were based on the following conditions: (1) schools were public; (2) contained at least 40 students aged 15 years; (3) had more than two teachers for students aged 15 years; and (4) had no more than 60% of students of the same gender. Eligible schools were categorized by size: (i) small (≤the median number of students in all schools of the study site); and (ii) large (≥the median number of students in all schools of the study site) [46]. Using a random number generator, schools were randomized according to SEYLE interventions and school size with respect to sociocultural factors, school environment and school system structure in each study site. 

Data were collected through structured questionnaires administered to adolescents within the school milieu. Representativeness, consent, participation and response rates of the sample are reported in a methodological analysis [47].

The present study was conducted in accordance with the Declaration of Helsinki, and the protocol was approved by the local Ethics Committee in each participating country (Project No. HEALTH-F2-2009-223091). Prior to participating in the study, both adolescents and parents provided their informed consent for participation. 

### 2.2. Measurements

PIU was assessed using Young’s Diagnostic Questionnaire (YDQ) [18]. The YDQ is an 8-item questionnaire assessing patterns of Internet usage that result in psychological or social impairment during the six-month period preceding data collection [48]. The eight items in the YDQ correspond to the six items in Griffiths’ components model and nine items in the diagnostic criteria of IGD in the DSM-5 [49,50]. Based on the YDQ score, ranging from 0–8, Internet users were categorized into three groups: adaptive Internet users (AIU) (scoring 0–2); maladaptive Internet users (MIU) (scoring 3–4); and pathological Internet users (PIU) (scoring ≥ 5) [51]. Moreover, hours online *per* day were measured using a single-item question in the structured questionnaire. 

Data on risk-behaviors were obtained by using questions from the Global School-Based Student Health Survey (GSHS) [52]. Developed by the World Health Organization (WHO) and collaborators, the GSHS is a school-based survey assessing health risk-behaviors among adolescents aged 13–17 years. This self-report questionnaire comprises items that correspond to the 10 leading causes of morbidity among adolescents and young people.

### 2.3. Individual Risk-Behaviors

Based on the GSHS, individual risk-behaviors were delineated into three categories: (i) substance use; (ii) sensation-seeking; (iii) and lifestyle characteristics. The ensuing individual risk-behaviors were coded as dichotomous variables.

#### 2.3.1. Substance Use

Substance use involved alcohol use, illicit drug use and use of tobacco. The variables were classified accordingly: (1) frequency of alcohol use: ≥2 times/week *vs.* ≤1 times/week; (2) number of drinks on a typical drinking day: ≥3 drinks *vs*. ≤2 drinks; (3) lifetime incidence of drinking to the point of drunkenness (alcohol intoxication): ≥3 times *vs*. ≤2 times; (4) lifetime incidence of having a hangover after drinking: ≥3 times *vs*. ≤2 times; (5) ever used drugs: yes/no; (6) ever used hashish or marijuana: yes/no; (7) ever used tobacco: yes/no; and (8) currently smoking cigarettes: ≥6/day *vs*. ≤5/day.

#### 2.3.2. Sensation-Seeking

Sensation-seeking comprised four items indicating risk-taking actions during the past twelve months: (1) driven in a vehicle by a friend who had been drinking alcohol; (2) ridden a skateboard or roller-bladed in traffic without a helmet and/or (3) pulled along a moving vehicle; and (4) gone to dangerous streets or alleys during night time. Response alternatives were yes/no in all four items. 

#### 2.3.3. Lifestyle Characteristics

Lifestyle characteristics included variables related to sleep, nutrition, physical activity and school attendance. Sleeping habits referred to the past six months: (1) feeling tired in the morning before school: ≥3 days/week *vs*. ≤2 days/week; (2) napping after school: ≥3 days/week *vs*. ≤2 days/week; and (4) sleeping: ≤6 hours/night *vs*. ≥7 hours/night. Nutrition referred to the past six months: (4) consuming fruits/vegetables: ≤1 time/week *vs*. ≥2 times/week; and (5) consuming breakfast before school: ≤2 days/week *vs*. ≥3 days/week. Physical activity referred to the past six months: (6) physical activity for at least 60 minutes during the past two weeks: ≤3 days *vs*. ≥4 days; and (7) playing sports on a regular basis: yes/no. School attendance comprised one item on the occurrence of unexcused absences from school during the past two weeks: ≥3 days *vs*. ≤2 days. 

### 2.4. Multiple Risk-Behaviors

The total number of risk-behaviors was calculated into a single variable and coded as an ordinal measure. Split-half reliability (*r_sb_* = 0.742) and internal consistency (α = 0.714) values indicated an acceptable level of homogeneity between items in the multiple risk-behavior measure.

## 3. Statistical Analyses

The prevalence of individual risk-behaviors among Internet user groups was calculated for males and females. To ascertain statistically-significant differences between group proportions, multiple pairwise comparisons using the two-sided z-test with Bonferroni adjusted *p*-values was performed. Extended analyses were conducted to test the effect of individual risk-behaviors on MIU and PIU using generalized linear mixed models (*GLMM*) with a multinomial logit link and full maximum likelihood estimation. In the GLMM analysis, MIU and PIU were entered as the outcome measures with AIU as the reference category, individual risk-behaviors were entered as Level 1 fixed effects, school as Level 2 random intercept and country as Level 3 random intercept. Variance components were used as the covariance structure for the random effects. To study the moderating effect of gender, interaction terms (gender * risk-behavior) were fitted into the regression model. Adjustments for age and gender were applied to relevant GLMM models. Odds ratios (*OR*) with 95% confidence intervals (*CI*) are reported for the respective models. 

In the analysis on multiple risk-behaviors, the mean (*M*) and standard error of the mean (*SEM*) were calculated for the different Internet user groups and stratified by gender. Box and whisker plots were used to illustrate these relationships. Statistical significance between multiple risk-behaviors and gender was assessed using independent samples *t*-test. One-way analysis of variance (*ANOVA*) with *post hoc* pairwise comparisons was employed to assess the statistical significance between multiple risk-behaviors and Internet user groups. 

A regression variable plot was conducted to elucidate the linear relationship between the number of hours online *per* day and the number of risk-behaviors among Internet user groups. All statistical tests were performed using IBM SPSS Statistics 23.0. A critical value of *p* < 0.05 was considered to be statistically significant.

## 4. Results

### 4.1. Characteristics of the Study Sample

Among the initial SEYLE sample of 12,395 adolescents, there were 464 (3.7%) subjects excluded due to missing data on relevant variables. This yielded a sample size of 11,931 school-based adolescents for the present study. The sample comprised 43.4% male and 56.6% female adolescents (M/F: 5179/6752) with a mean age of 14.89 ± 0.87 years. The prevalence of MIU was significantly higher among females (14.3%) compared to males (12.4%), whereas PIU was significantly higher among males (5.2%) than females (3.9%) (*χ²* (2, 11928) = 19.92, *p* < 0.001).

### 4.2. Prevalence of Risk-Behaviors 

Table 1 describes the prevalence of risk-behaviors stratified by Internet user group. The average prevalence rates among Internet user groups (AIU, MIU and PIU) were 16.4%, 24.3% and 26.5% for substance use (alcohol use, illicit drug use and tobacco use); 19.0%, 27.8% and 33.8% for sensation-seeking behaviors (risk-taking actions); and 23.8%, 30.8% and 35.2% for lifestyle characteristics (poor sleeping habits, poor nutrition, physical inactivity and truancy), respectively. Prevalence within MIU and PIU groups was significantly higher compared to the AIU group in all risk categories (substance use, sensation-seeking and lifestyle characteristics). With the exception of five subcategories, pairwise comparisons showed that prevalence rates did not significantly differ between MIU and PIU groups. 

### 4.3. Multiple Risk-Behaviors 

**Figure 1:**
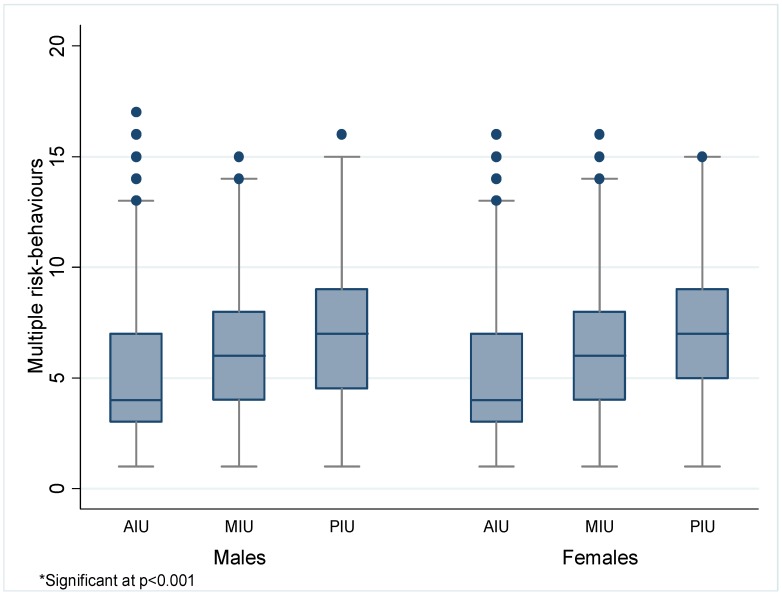
Box and whisker plot of multiple risk-behaviors among adaptive Internet users (AIU), maladaptive Internet users (MIU) and pathological Internet users (PIU) stratified by gender *.

Results showed that 89.9% of adolescents in the PIU group reported multiple risk-behaviors. The one-way ANOVA test revealed that the mean rate of multiple risk-behaviors significantly increased from adaptive use (M = 4.89, SEM = 0.02) to maladaptive use (M = 6.38, SEM = 0.07) to pathological use (M = 7.09, SEM = 0.12) (F (2, 11928) = 310.35, *p* < 0.001). This trend was virtually equivalent for males and females (Figure 1).

Moreover, no statistical difference between genders in both MIU (*t* (1608) = 0.529, *p* = 0.597) and PIU (*t* (526) = 1.92, *p* = 0.054) groups were observed (Table 2). It should be noted, however, that the *p*-value for the PIU group was relatively close to reaching statistical significance (*p* = 0.054).

**Figure 2:**
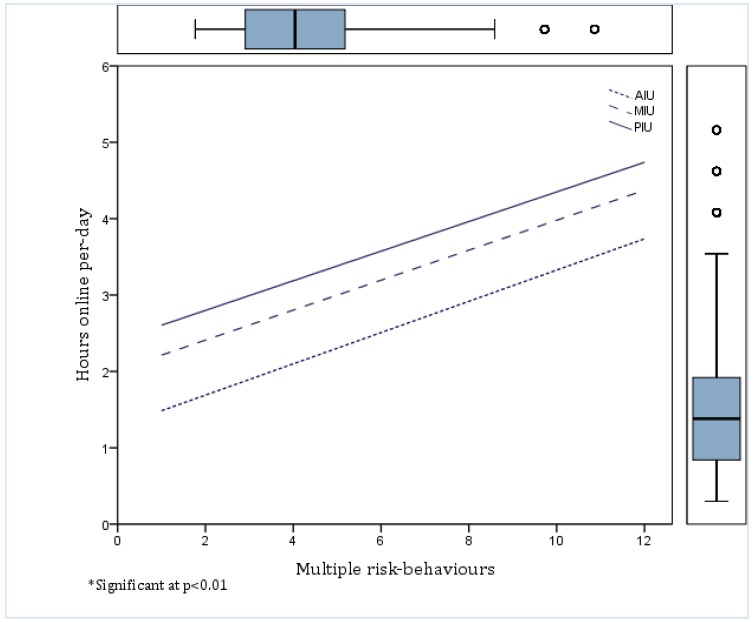
Linear relationship between the number of hours online per day and the number of risk-behaviors among AIU, MIU and PIU groups *.

The regression variable plot exhibited a clear linear relationship between the number of hours online per day and the number of risk-behaviors in adolescents. This trend was comparatively identical between Internet user groups (Figure 2).

### 4.4. GLMM Analysis of the Association between Risk-Behaviors, MIU and PIU

Risk-behaviors that were significantly associated with MIU were also significantly associated with PIU, with the exception of three subcategories noted within risk-taking actions and truancy (Table 3). The GLMM analysis showed that all subcategories of poor sleeping habits significantly increased the relative odds of PIU with effect sizes ranging from *OR* = 1.45 to *OR* = 2.17. Significant associations were observed between risk-taking actions and PIU with effect sizes ranging from *OR* = 1.55 to *OR* = 1.73. Moreover, odds ratios for single subcategories within the tobacco use (*OR* = 1.41), poor nutrition (*OR* = 1.41) and physical inactivity (*OR* = 1.39) domains were statistically significant.

### 4.5. Gender Interactions

The analysis on gender interactions revealed that the association between risk-taking actions, poor sleeping habits and PIU was significantly higher in females, whereas the association between truancy, poor nutrition and PIU was significantly higher in males (Table 3).

## 5. Discussion

### 5.1. Prevalence of Risk-Behaviors 

The present study sought to examine the relationship between PIU and risk-behaviors. Results showed that the prevalence of risk-behaviors was significantly higher among pathological users compared to adaptive users with some variations between genders. The highest prevalence observed among maladaptive and pathological users was poor sleeping habits followed by tobacco use. These estimations are considerably higher compared to prevalence rates reported in studies conducted outside the EU, namely in the Asia and Pacific regions [53,54]. One plausible explanation could be related to the variations observed at the ecological level (e.g., penetration rates) among these respective regions. Statistics show that the European region has the highest Internet penetration rate (78%) worldwide. European rates are more than double compared to those depicted in the Asia and Pacific regions (36%) [55]. The actual role penetration rates have on influencing the prevalence of PIU remains ambiguous; thus, future efforts examining this relationship would be of great value for explaining this connection.

### 5.2. Substance Use 

The characteristics between risk-behaviors and addictive behaviors are highly overlapping. This is perhaps most evident with substance use. Substance use is often classified as a risk-behavior; however, it is also an antecedent of substance abuse. If high-risk behaviors share similar underlying mechanisms, then having one problem behavior may lower the threshold to developing other problem behaviors. This assertion is corroborated by evidence-based research demonstrating a high level of interconnectedness between various risk-behaviors [56]. Based on this concept, it is plausible to assume that adolescents with pre-existing risk-behaviors are likely to have a higher risk of PIU compared to adolescents without risk-behaviors. 

### 5.3. Sensation-Seeking 

In line with the foregoing research [57], results showed that the majority of risk-taking actions within the sensation-seeking category were significantly associated with PIU. Sensation-seeking is a personality trait associated with deficiencies in self-regulation and deferred gratification [58]. These attributes among youth are often related to a perceptual predisposition of an ‘optimistic bias effect’ in which adolescents are more likely to discount risks for themselves, while overestimating risks for others [59]. Adolescents exhibiting these deflecting traits are likely to have a higher propensity for behavioral problems. 

### 5.4. Lifestyle Characteristics 

Poor sleeping habits proved to be the strongest factors related to PIU. This is likely due to a displacement effect of sleep for online activities. There are certain online activities that explicitly induce users to stay online longer than anticipated. A study on massively multiplayer online role-playing games (MMORPG) indicated that users are enticed to stay online longer in order to follow the progressive storyline of their online character [60]. Excessive use of social networking sites has also emerged in recent years, denoting both an increase in time spent online and negative correlations with real-life social interactions [61,62]. Studies show that adolescents excessively using the Internet have the propensity for developing sleeping disorders as a result of their extended time online [63,64]. The chronic displacement of sleep for online activities could lead to sleep deprivation, which is known to cause severe adverse effects on social, psychological and somatic functioning. 

Disturbances in regulated sleep patterns could also be a mediating factor in the relationship between truancy and maladaptive use of the Internet. Adolescents engaging in online activities to an excessive degree could run the risk of disrupting their natural order of sleep. Evidence shows that increased sleep latency and decreased rapid eye movement sleep (REM-sleep) are significantly associated with excessive Internet use [65], while subjective insomnias and parasomnias are linked with truancy [66]. Sleep disorders have pronounced effects on daytime functioning and academic achievement. This could cause adolescents to become disinterested in school, thereby increasing the risk of school refusal and chronic absenteeism [66].

Poor nutrition and physical inactivity were shown to be significantly associated with PIU. Adolescents who spend longer hours online potentially navigate towards unhealthier foods. It is postulated that online gamers drink high-caffeinated energy drinks and eat high-sugar snacks to increase alertness for online gaming [67]. Subsequently, these factors could make online gamers more inclined to sedentary behaviors compared to non-gamers. Moreover, there is an extensive loyalty among gamers, particularly those who displace food, personal hygiene and physical activity, in order to continue with online games [68]. This could pose serious health-risks and may lead to severe psychosomatic symptoms.

### 5.5. Multiple Risk-Behaviors

Risk-behaviors were ascertained to be concurrent in nature, with 89.9% of adolescents in the PIU group reporting multiple risk-behaviors. These results are in line with Jessor’s theory on problem behavior [69,70]. The problem behavior theory is a psychosocial model that attempts to explain behavioral outcomes in adolescents. It consists of three conceptual systems based on psychosocial components: personality system, perceived environmental system and behavior system. In the latter system, risk-behavior structures (e.g., alcohol use, tobacco use, delinquency and deviancy) tend to co-occur and cluster into a general ‘risk-behavior syndrome’ [71]. According to Jessor, these problem behaviors often stem from adolescents’ assertion of independence from parents and societal influences. 

Adolescents struggling for autonomy could, in part, account for the significant linear trend noted between hours online per day and multiple risk-behaviors. This trend was comparatively identical across all Internet user groups. These findings are highly relevant, as they suggest that excessive hours online in itself can increase the number of risk-behaviors for all adolescents and not only those diagnosed with PIU. Excessive hours online could also be a moderating factor in the relationship between PIU and risk-behaviors; however, further research exploring this relationship is necessitated.

### 5.6. Gender Interactions

The analysis on gender interactions showed that significant associations observed between risk-behaviors and PIU were evenly distributed between males and females. This is somewhat contradictory to previous research, which typically shows that PIU and risk-behaviors are specific to the male gender. This gender shift could be an indication that the gender gap for risk-behaviors may be narrowing among European adolescents. 

From another perspective, the relationship between gender and risk-behaviors could be mediated by a third factor, such as psychopathology. In a large, gender-based study of adolescents (n = 56,086) aged 12–18 years, prevalence rates for PIU were estimated to be 2.8% among the total sample with significantly higher rates observed in males (3.6%) compared to females (1.9%) [72]. The respective study noted that females with emotional issues, such as subjective unhappiness or depressive symptoms, have a significantly higher PIU prevalence than males with similar emotional symptoms. Gender-based studies scrutinizing the effect of gender interactions on PIU are an essential prerequisite for the future direction of PIU research.

### 5.7. Griffiths’ Components Model 

Griffiths’ components model of addiction [30] hypothesizes that behavioral addictions (e.g., PIU) and substance-related addictions advance via similar biopsychosocial processes and share numerous physiognomies. The addiction criteria of the respective six core components in this model are (1) salience, (2) mood modification, (3) tolerance, (4) withdrawal, (5) conflict and (6) relapse. Kuss *et al.* [73] assessed the components model of addiction in two independent samples (n = 3105 and n = 2257). Results showed that the components model of PIU fit the data very well in both samples. 

In the present study, the YDQ measure was utilized to assess and detect adolescents with maladaptive and pathological risks related to their Internet use and online behaviors. As the YDQ measure comprises all six addiction criteria stipulated in Griffiths’ components model, the validity of the outcomes reported in this study is supported by this theoretical framework.

### 5.8. Strengths and Limitations

The large, representative, cross-national sample is a major strength of this study. The homogenous methodology and standardized procedures utilized in all countries increases the validity, reliability and comparability of the data. To the extent of our knowledge, the geographic area in Europe was the largest ever used to conduct a study on PIU and risk-behaviors. 

There are also some limitations of the study. Self-reported data are prone to recall and social desirability biases, which is likely to vary between countries and cultures. The cross-sectional design is unable to account for temporal relationships, thus causality could not be determined. In the GSHS measure, the subcategories of risk-taking actions only represent a part of sensation-seeking behaviors; thus, caution should be used when interpreting the results. 

## 6. Conclusions

A significantly increasing prevalence rate across AIU, MIU and PIU groups was observed in all risk categories (substance use, sensation-seeking and lifestyle characteristics). Adolescents reporting poor sleeping habits and risk-taking actions showed the strongest associations with PIU, followed by tobacco use, poor nutrition and physical inactivity. The significant association observed between PIU and risk-behaviors, combined with a high rate of co-occurrence, underlines the importance of considering PIU when screening, treating or preventing high-risk behaviors in adolescents. 

Among adolescents in the PIU group, 89.9% were characterized as having multiple risk-behaviors. Thus, efforts should target adolescents who excessively use the Internet, as a significant linear trend was observed between hours online per day and multiple risk-behaviors. This trend was similar across all Internet user groups indicating that excessive hours online in itself is an important factor for risk-behaviors. These findings need to be replicated and further explored before ascertaining their theoretical implications.

## Figures and Tables

**Table 1:** Prevalence of risk-behaviors among adolescents stratified by gender and Internet user group ^1,2a–c^.

Prevalence of Risk-Behaviors among Adolescents			Adaptive Use			Maladaptive Use			Pathological Use		
			Male	Female	Total	Male	Female	Total	Male	Female	Total
RCAT	Risk-Behaviors	Subcategories	%	%	%	%	%	%	%	%	%
**Substance use**	**Alcohol Use**	Drinking alcohol ≥2 times/week	10.6 ^a^	5.7 ^b^	7.9 ^a^	15.6 ^a^	9.4 ^b^	11.9 ^b^	16.4 ^a^	11.2 ^a^	13.8 ^b^
		≥3 drinks on a typical drinking day	24.0 ^a^	20.0 ^b^	21.8 ^a^	31.3 ^a^	31.4 ^a^	31.4 ^b^	34.3 ^a^	38.1 ^a^	36.2 ^b^
		Alcohol intoxication ≥3 times/lifetime	16.1 ^a^	10.5 ^b^	13.0 ^a^	25.1 ^a^	18.1 ^b^	20.9 ^b^	25.4 ^a^	25.0 ^a^	25.2 ^b^
		Hangover after drinking ≥3 times/lifetime	8.5 ^a^	6.0 ^b^	7.1 ^a^	15.6 ^a^	9.4 ^b^	11.9 ^b^	13.8 ^a^	15.4 ^a^	14.6 ^b^
	**Illicit Drug Use**	Have used drugs during lifetime	6.1 ^a^	3.7 ^b^	4.8 ^a^	11.1 ^a^	5.7 ^b^	7.8 ^b^	7.5 ^a^	8.8 ^a^	8.1 ^b^
		Have used hashish or marijuana during lifetime	10.6 ^a^	6.7 ^b^	8.4 ^a^	15.4 ^a^	10.5 ^b^	12.5 ^b^	15.3 ^a^	15.2 ^a^	15.3 ^b^
	**Tobacco Use**	Have smoked cigarettes during lifetime	41.2 ^a^	44.0 ^b^	42.8 ^a^	55.8 ^a^	61.4 ^b^	59.1 ^b^	60.1 ^a^	65.8 ^a^	62.9 ^b^
		Currently smoking ≥6 cigarettes/day	26.1 ^a^	31.2 ^b^	29.0 ^a^	31.6 ^a^	43.5 ^b^	38.8 ^b^	31.3 ^a^	41.2 ^b^	36.2 ^b^
**Sensation seeking**	**Risk-Taking Actions**	Driven in a vehicle by a friend who has been drinking alcohol during lifetime	16.5 ^a^	14.1 ^b^	15.2 ^a^	24.1 ^a^	20.8 ^a^	22.1 ^b^	28.7 ^a^	29.6 ^a^	29.2 ^c^
		Ridden skateboard or roller-blades in traffic without a helmet during lifetime	31.1 ^a^	24.2 ^b^	27.2 ^a^	37.7 ^a^	36.1 ^a^	36.7 ^b^	35.4 ^a^	43.5 ^a^	39.4 ^b^
		Pulled along a moving vehicle during lifetime	7.8 ^a^	2.3 ^b^	4.7 ^a^	12.8 ^a^	4.9 ^b^	8.0 ^b^	19.8 ^a^	6.5 ^b^	13.3 ^c^
		Gone to dangerous streets or alleys at night-time during lifetime	33.8 ^a^	25.0 ^b^	28.9 ^a^	47.7 ^a^	42.3 ^b^	44.4 ^b^	53.0 ^a^	53.8 ^a^	53.4 ^c^
**Lifestyles Characteristics**	**Truancy**	Unexcused absences from school ≥3 days/two-weeks	3.9 ^a^	2.2 ^b^	3.0 ^a^	9.0 ^a^	4.6 ^b^	6.4 ^b^	11.9 ^a^	5.8 ^b^	8.9 ^b^
	**Poor Sleeping Habits**	Feeling tired in the morning before school ≥3 days/week	52.7 ^a^	57.4 ^b^	55.4 ^a^	70.1 ^a^	74.4 ^a^	72.7 ^b^	71.6 ^a^	82.7 ^b^	77.1 ^b^
		Napping after school ≥3 days/week	21.1 ^a^	19.0 ^a^	19.8 ^a^	26.6 ^a^	24.1 ^a^	25.1 ^b^	36.5 ^a^	23.7 ^b^	30.7 ^b^
		Sleeping ≤6 h/night	11.8 ^a^	15.0 ^b^	13.6 ^a^	16.7 ^a^	26.3 ^b^	22.5 ^b^	23.9 ^a^	35.8 ^b^	29.7 ^c^
	**Poor Nutrition**	Consuming fruits and vegetables ≤1 time/week	16.9 ^a^	11.6 ^b^	13.9 ^a^	24.8 ^a^	18.1 ^b^	20.7 ^b^	32.5 ^a^	20.0 ^b^	26.3 ^c^
		Consuming breakfast before school ≤2 days/week	31.0 ^a^	41.1 ^b^	36.7 ^a^	36.1 ^a^	49.1 ^b^	43.9 ^b^	41.0 ^a^	56.5 ^b^	48.7 ^b^
	**Physical Inactive**	Physical activity ≤3 days/two-weeks	13.2 ^a^	22.4 ^b^	18.4 ^a^	18.5 ^a^	23.7 ^b^	21.6 ^b^	20.9 ^a^	26.5 ^a^	23.7 ^b^
		Does not play sport(s) on a regular basis	20.2 ^a^	37.2 ^b^	29.8 ^a^	24.8 ^a^	39.2 ^b^	33.4 ^b^	28.0 ^a^	44.6 ^b^	36.2 ^b^

^1^ N = 11,931 (AIU = 9793 (M/F: 4269/5524), MIU = 1610 (M/F: 642/968), PIU = 528 (M/F: 268/260)); RCAT = risk categories; ^2a^ Gender values (a and b) in the same row and sub-table not sharing the same subscript indicate significant differences at *p* < 0.05. ^2b^ Total column values (a,b,c) in the same row not sharing the same subscript indicate significant differences between Internet user groups at *p* < 0.05. ^2c^ Multiple pairwise comparisons were assessed using the two-sided z-test of proportions with Bonferroni-corrected *p*-values.

**Table 2:** Independent samples *t*-test of multiple risk-behaviors and gender by Internet user group ^1–3^.

Internet User Groups	Multiple Risk-Behaviors									
	Homogeneity of variances									
	F	*p*-value	Gender	Mean	SEM	*t*	df	*p*-value	Mean Difference	SE Difference
**Adaptive use**	48.254	<0.001	Male	4.87	0.04	0.943	9791	0.345	0.055	0.058
			Female	4.92	0.03					
**Maladaptive use**	2.238	0.135	Male	6.33	0.11	0.529	1608	0.597	0.077	0.146
			Female	6.41	0.09					
**Pathological use**	0.060	0.806	Male	6.85	0.18	1.928	526	0.054	0.492	0.255
			Female	7.34	0.18					

^1^ N = 11,931 (AIU = 9793 (M/F: 4269/5524), MIU = 1610 (M/F: 642/968), PIU = 528 (M/F: 268/260). ^2^ Model abbreviations include the F-statistic (F), standard error of the mean (SEM), standard error (SE), *t*-statistic (*t*) and degrees of freedom (df). ^3^ Levene’s test for equality of variances was performed to assess homogeneity (*p* > 0.05 indicates equal variances).

**Table 3:** Generalized linear mixed model (GLMM) of the association between individual risk-behaviors, maladaptive use and pathological use with an extended analysis on gender interactions ^1–4^.

RCAT ^a^	Risk-Behaviors	Subcategories	Maladaptive Use ^b^			Pathological Use ^c^			Gender
			OR	95% CI	*p*-Value	OR	95% CI	*p*-Value	Interactions ^d^
**Substance Use**	**Alcohol Use**	Drinking alcohol ≥2 times/week	1.00	0.82–1.22	0.943	0.88	0.64–1.20	0.426	n.s.
		≥3 drinks on a typical drinking day	1.07	0.92–1.24	0.336	1.25	0.99–1.59	0.060	n.s.
		Alcohol intoxication ≥3 times/lifetime	1.03	0.85–1.24	0.718	1.07	0.80–1.43	0.646	n.s.
		Hangover after drinking ≥3 times/lifetime	1.01	0.82–1.26	0.872	0.98	0.71–1.36	0.920	n.s.
	**Illicit Drug Use**	Have used drugs during lifetime	1.07	0.82–1.41	0.583	0.81	0.53–1.22	0.322	n.s.
		Have used hashish or marijuana during lifetime	0.85	0.68–1.07	0.178	0.93	0.67–1.30	0.695	n.s.
	**Tobacco Use**	Have smoked cigarettes during lifetime	**1.29**	1.08–1.55	0.004	**1.41**	1.06–1.88	0.018	Male *
		Currently smoking ≥6 cigarettes/day	1.06	0.90–1.25	0.456	0.92	0.71–1.20	0.575	n.s.
**Sensation Seeking**	**Risk-Taking Actions**	Driven in a vehicle by a friend who has been drinking alcohol during lifetime	1.14	0.99–1.32	0.060	1.55	1.24–1.94	<0.001	Female *
		Ridden skateboard or roller-blades in traffic without a helmet during lifetime	**1.18**	1.04–1.34	0.008	**1.07**	0.87–1.32	0.480	Female **
		Pulled along a moving vehicle during lifetime	1.24	0.99–1.55	0.055	1.64	1.20–2.24	0.002	Male ***
		Gone to dangerous streets or alleys at night-time during lifetime	**1.46**	1.29–1.66	<0.001	**1.73**	1.41–2.14	<0.001	Female ***
**Lifestyles Characteristics**	**Truancy**	Unexcused absences ≥3 days/two-weeks	**1.39**	1.08–1.79	0.010	**1.22**	0.85–1.75	0.268	Male *
	**Poor Sleeping Habits**	Feeling tired in the morning before school ≥3 days/week	**1.77**	1.57–2.01	<0.001	**2.17**	1.74–2.72	<0.001	Female ***
		Napping after school ≥3 days/week	**1.27**	1.12–1.36	0.024	**1.45**	1.19–1.77	<0.001	Male ***
		Sleeping ≤6 hrs/night	**1.29**	1.12–1.48	<0.001	**1.80**	1.45–2.24	<0.001	Female ***
	**Poor Nutrition**	Consuming fruits and vegetables ≤1 time/week	**1.34**	1.16–1.55	<0.001	**1.41**	1.13–1.76	0.002	Male ***
		Consuming breakfast before school ≤2 days/week	1.03	0.92–1.16	0.566	1.17	0.96–1.42	0.114	n.s.
	**Physical Inactivity**	Physical activity ≤3 days/two-weeks	**1.18**	1.02–1.36	0.024	**1.39**	1.10–1.76	0.006	n.s.
		Does not play sport(s) on a regular basis	1.02	0.90–1.16	0.715	1.12	0.91–1.38	0.264	n.s.

^1^ N = 11,931 (AIU = 9793, MIU = 1610, PIU = 528). ^2^ Adaptive use is the reference category. ^3^ Outcomes are presented as an odds ratio (*OR*) with 95% confidence intervals (95% CI) and *p*-values. ^4^ Models are adjusted for age and gender. ^a^ RCAT = risk categories. ^b^ Random-effects parameter (country * school) = 0.133; CI: 0.008–0.203, *p* < 0.001. ^c^ Random-effects parameter (country * school) = 0.356; CI: 0.230–0.550, *p* < 0.001. ^d^ Interaction terms (gender * risk-behavior). n.s*.* = not significant; * *p* < 0.05; ** *p* < 0.01; *** *p* < 0.001.

## Abbreviations

The following abbreviations are used in this manuscript:

SEYLE: Saving and Empowering Young Lives in Europe
YRBSS: Youth Risk Behavior Surveillance System
GSHS: Global School-Based Student Health Survey
YDQ: Young’s Diagnostic Questionnaire
GLMM: Generalized linear mixed models
ANOVA: One-way analysis of variance
PIU: Pathological Internet use
MIU: Maladaptive Internet use
AIU: Adaptive Internet use
CI: Confidence intervals
SEM: Standard error of the mean
M: Mean
